# Reversal of the diabetic bone signature with anabolic therapies in mice

**DOI:** 10.1038/s41413-023-00261-0

**Published:** 2023-04-19

**Authors:** Silvia Marino, Nisreen Akel, Shenyang Li, Meloney Cregor, Meghan Jones, Betiana Perez, Gaston Troncoso, Jomeeka Meeks, Scott Stewart, Amy Y. Sato, Intawat Nookaew, Teresita Bellido

**Affiliations:** 1 Department of Physiology and Cell Biology, University of Arkansas for Medical Sciences, Little Rock, AR USA; 2 Central Arkansas Veterans Healthcare System, John L. McClellan Little Rock, Little Rock, AR USA; 3 Department of Biostatistics, University of Arkansas for Medical Sciences, Little Rock, AR USA; 4 Department of Biomedical Informatics, University of Arkansas for Medical Sciences, Little Rock, AR USA; 5 Winthrop P. Rockefeller Cancer Institute, University of Arkansas for Medical Sciences, Little Rock, AR USA

**Keywords:** Bone quality and biomechanics, Pathogenesis

## Abstract

The mechanisms underlying the bone disease induced by diabetes are complex and not fully understood; and antiresorptive agents, the current standard of care, do not restore the weakened bone architecture. Herein, we reveal the diabetic bone signature in mice at the tissue, cell, and transcriptome levels and demonstrate that three FDA-approved bone-anabolic agents correct it. Diabetes decreased bone mineral density (BMD) and bone formation, damaged microarchitecture, increased porosity of cortical bone, and compromised bone strength. Teriparatide (PTH), abaloparatide (ABL), and romosozumab/anti-sclerostin antibody (Scl-Ab) all restored BMD and corrected the deteriorated bone architecture. Mechanistically, PTH and more potently ABL induced similar responses at the tissue and gene signature levels, increasing both formation and resorption with positive balance towards bone gain. In contrast, Scl-Ab increased formation but decreased resorption. All agents restored bone architecture, corrected cortical porosity, and improved mechanical properties of diabetic bone; and ABL and Scl-Ab increased toughness, a fracture resistance index. Remarkably, all agents increased bone strength over the healthy controls even in the presence of severe hyperglycemia. These findings demonstrate the therapeutic value of bone anabolic agents to treat diabetes-induced bone disease and suggest the need for revisiting the approaches for the treatment of bone fragility in diabetes.

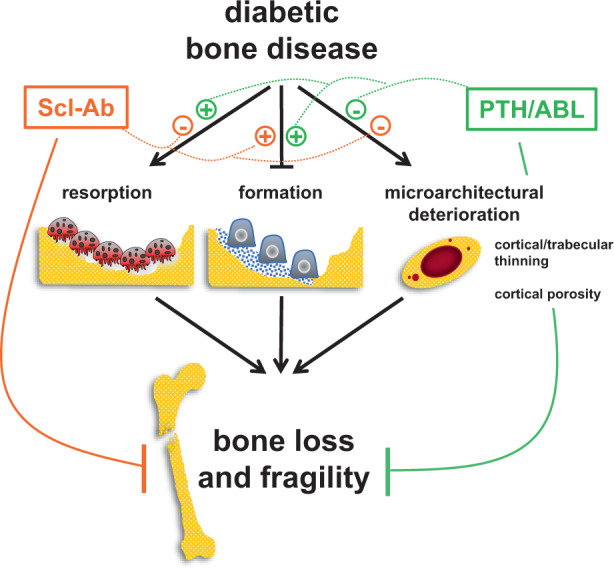

## Introduction

The global prevalence of diabetes mellitus (DM) around the world is high with more than 500 million adults living with the disease (1 in 10, ages 20–79), and it is estimated to increase to 783 million by 2045.^1^ The American Diabetes Association declared DM a nationwide epidemic, being the 7^th^ leading cause of death in the US. In 2019, ~11% of Americans had DM and ~50% of Americans 65 years and older had prediabetes. The disease impacts all tissues and organ systems, accounting for substantial morbidity and mortality. In 2021, over 400 billion dollars were spent in the US on DM-related health-care costs. DM is characterized by high glucose levels due to insufficient production or inefficient utilization of insulin in Type 1 (T1-DM) or T2-DM, respectively.^2–4^ Regardless of the cause, a major complication of DM is the bone disease and increased fragility with a 32% increased risk of bone fractures in diabetic patients compared to non-diabetics .^5,6,7^

The mechanisms underlying DM-induced bone disease are complex and uncertain. Patients with T1-DM can exhibit low bone mass, measured as bone mineral density (BMD), whereas those with T2-DM can exhibit normal or even increased BMD. Yet, bone fragility is increased potentially due to accumulation of advanced glycation end products (AGEs) in collagen and other bone matrix proteins, which decreases bone toughness and resistance to fracture.^8^ Cortical bone micro-architectural deterioration secondary to reduced thickness and increased porosity is another feature of diabetic bone.^6,9,10^ Thus, bone fragility in DM is associated with deteriorated intrinsic as well as extrinsic properties of bone accompanied or not by changes in BMD.

A common feature of the bone disease with T1 or T2-DM is reduced osteoblast number and function and low bone formation.^5^ However, the standard of care are anti-resorptive agents, bisphosphonates or denosumab, which stop bone loss but do not increase bone formation, thus failing to repair the deteriorated bone architecture. In the current study, we investigated the effectiveness of the three agents with bone anabolic properties approved by the FDA for the treatment of osteoporosis: teriparatide/parathyroid hormone 1-34 (PTH), abaloparatide/PTH-related peptide 1-34 (ABL), and romosozumab/anti-sclerostin antibody (Scl-Ab)], in restoring the weakened bone structure using a preclinical murine model of established T2-DM.

The current study reveals the signature of the diabetic bone at the tissue, cell, and transcriptome levels and demonstrates that all anabolic agents reverse it, rebuilding the bone lost with DM, increasing bone formation, correcting the elevated cortical porosity, and restoring bone strength. However, whereas PTH/ABL increased resorption, the Scl-Ab decreased it leading to further bone gain. In addition, all agents restored bone area, increased cortical thickness and corrected the weakened structural properties of diabetic bone. Furthermore, ABL and Scl-Ab increased toughness, a measure of the energy absorbed by bone before breaking associated with fracture risk. Our findings demonstrate the efficacy of increasing bone formation (independently on the effects on resorption) to restore the damaging effects of diabetes on bone mass and structure, and to increase bone strength.

## Results

### PTH and ABL restored the bone lost with T2-DM

**Fig. 1:**
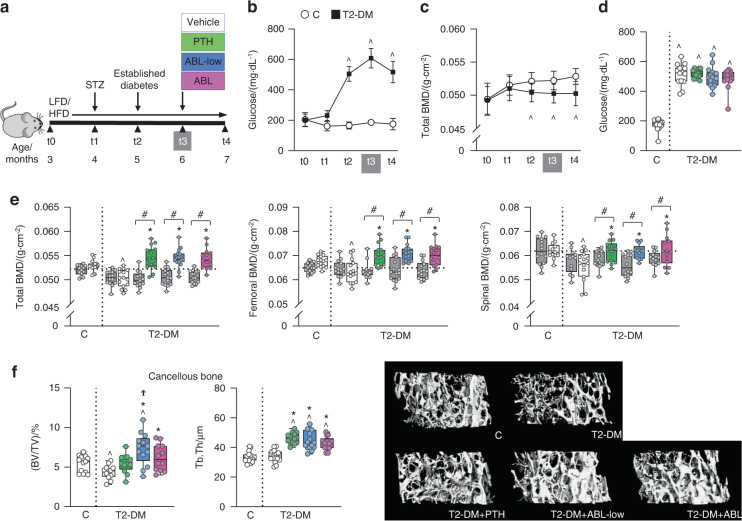
PTH and both doses of ABL restored BMD and increased cancellous bone in diabetic mice. **a** Study design depicting the preclinical T2-DM model. Male C57BL/6 mice were fed a low-fat diet (LFD) or a high fat diet (HFD) starting at t0 until the end of the experiment. At t1, HFD-fed mice were injected with streptozotocin (STZ) (T2-DM) and LFD-fed mice, with buffer (C). At t2, blood glucose was measured to confirm DM, and following an additional month to fully develop the bone disease at t3, mice were administered with vehicle, PTH or ABL daily for 4 weeks (t4). Longitudinal analysis showing the effect of DM on **b** blood glucose, and **c** total bone mineral density (BMD). **d** Glucose levels after treatment (at t4). **e** Total, femoral and spinal BMD before treatment (at t3, grey bars) and after treatment (at t4) with vehicle (white bars), PTH (green bars) or ABL (blue and pink bars). **f** Micro-CT analysis of femur cancellous bone: trabecular bone volume/tissue volume (BV/TV) and trabecular thickness (Tb.Th), after treatment with PTH or ABL and representative images. *n* = 12–15 mice per group. Data are presented as box & whisker plots where each dot represents a mouse. ^*P* < 0.05 versus C mice by one-way ANOVA with post hoc Dunnet’s correction; *P < 0.05 versus T2-DM mice treated with vehicle; and ^#^*P* < 0.05 versus t3, by one-way ANOVA with post hoc Tukey’s correction

The bone anabolic agents were tested in a model of T2-DM induced by a combination of high fat diet (HFD) and streptozotocin (STZ) in skeletally mature male C57BL/6 J mice (Fig. 1a). HFD/STZ (T2-DM) caused persistent overt hyperglycemia with blood glucose > 250 mg·dL^−1^ compared to non-diabetic mice fed low fat diet (LFD, C mice), which was first detected 4 weeks after initiating the STZ injections (at t2) and remained elevated for the entire study (Fig. 1b). Body weight was increased in T2-DM mice compared to C after 4 weeks of HFD (at t1) (Fig. S1a), due to a gain in fat mass. T2-DM caused significant reduction in BMD as quantified by longitudinal DEXA analysis, confirming the development of bone disease (Fig. 1c and Fig. S1b). Decreased BMD was first detected in the spine at t1 and in total body at t2, and remained reduced compared to C throughout the study, whereas the decreased femoral BMD was detected at the end of the study. Four weeks of treatment with equimolar doses (24 pmol·g^−1^ per day) of PTH (100 μg·kg^−1^ per day) or ABL (95 μg·kg^−1^ per day) restored the bone lost caused by T2-DM at all sites (Fig. 1e). Similar increases in BMD were induced by half dose of ABL (ABL-low, 12 pmol·g^−1^ per day or 47.5 μg·kg^−1^ per day). Blood glucose, body weight, and fat mass remained elevated in T2-DM mice receiving PTH or ABL (Fig. 1d and Fig. S1c).

### PTH and ABL corrected the architectural deterioration induced by diabetes in cancellous and cortical bone and increased bone strength

T2-DM mice exhibited a reduction in bone volume/total volume (BV/TV) in cancellous bone of the distal femur measured by micro-CT compared to C mice, which was corrected by both doses of ABL and by PTH (Fig. 1f and Table S1). Further, ABL increased bone over healthy C and PTH did not. However, PTH and both doses of ABL were equally potent in increasing trabecular thickness (Tb.Th) in T2-DM mice, reducing trabecular number (Tb.N) and increasing trabecular separation (Tb.Sp), suggesting trabecular fusion. In addition, ABL significantly increased trabecular connectivity density (Conn D.). In cancellous bone of the spine (L6), PTH and ABL increased BV/TV to a similar extent (Table S2). PTH and ABL also increased vertebral trabecular thickness and reduced the structural model index (SMI), suggesting geometrical benefits provided by more plate-like structures versus more rod-like structures.

**Fig. 2:**
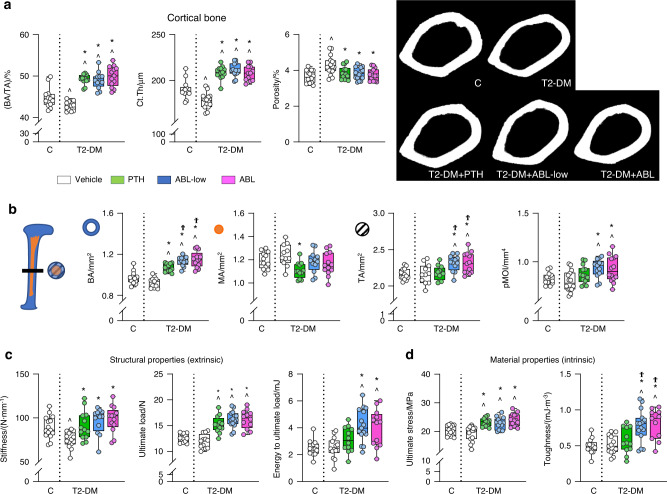
PTH and ABL corrected the cortical architectural deterioration induced by diabetes and increased bone strength. Micro-CT analysis of femur cortical bone microarchitecture and 3-point bending analysis of bone strength after treatment with PTH or ABL. **a** Cortical bone area/tissue area (BA/TA), thickness (Ct.Th) and porosity and representative images. **b** Bone area (BA), medullary area (MA), total area (TA) and polar moment of inertia (pMOI). **c** Bone structural properties (stiffness, ultimate load, and energy to ultimate load) and **d** bone material properties (ultimate stress and toughness). *n* = 12–15 mice per group. Data are presented as box & whisker plot where each dot represents a mouse. ^*P* < 0.05 versus C mice by one-way ANOVA with post hoc Dunnet’s correction; *P < 0.05 versus T2-DM mice treated with vehicle and ^Ϯ^*P* < 0.05 versus T2-DM mice treated with PTH by one-way ANOVA with post hoc Tukey’s correction

In cortical bone, T2-DM mice exhibited decreased cortical thickness (Ct.Th) and BA/TA (Fig. 2a). PTH and ABL were equally potent in restoring cortical thickness and bone area/total area (BA/TA) and further increasing these indexes as well as bone area (BA) over C (Fig. 2b). ABL increased bone area even further compared to PTH. In addition, T2-DM increased porosity at the femoral mid-diaphysis and PTH and ABL corrected it (Fig. 2a and Table S1). Restoration of cortical thickness by PTH vs ABL might result from bone surface-specific mechanisms, as PTH reduced marrow area (MA) whereas ABL increased tissue area (TA). Consistently, ABL but not PTH increased the polar moment of inertia (pMOI), resulting from a change in bone geometry leading to increased strength. Further, T2-DM induced marked changes in the geometry of the vertebra (L6), which were overall improved by the anabolic agents (Table S2). Specifically, T2-DM bones exhibited decreased vertebral cross-sectional area (CSA) which was corrected by PTH or ABL treatments. In addition, T2-DM decreased vertebral cortical thickness, which was corrected by ABL but not PTH.

T2-DM deteriorated the structural properties of the femur without affecting its material properties, quantified by three-point bending, and the anabolic agents corrected the structural changes and improved both structural and material properties (Fig. 2c, d and Table S1). Specifically, T2-DM bones exhibited lower stiffness, which was increased by PTH and ABL. The treatments also increased ultimate force and only ABL increased energy to ultimate load. Remarkably, PTH and ABL increased ultimate stress and ABL also increased material toughness above C levels. No differences in structural or mechanical properties of the vertebral bone were detected in T2-DM (Table S2). However, PTH increased ultimate force, PTH and ABL increased energy to ultimate load, and ABL increased energy to yield, over healthy C.

In summary, PTH and ABL restored and further increased cancellous and cortical bone volume and improved microarchitecture and extrinsic and intrinsic bone properties despite the ongoing hyperglycemic status.

### PTH and ABL converted the low bone remodeling disease in diabetes into a high bone remodeling condition with bone gain, and ABL was more potent

**Fig. 3:**
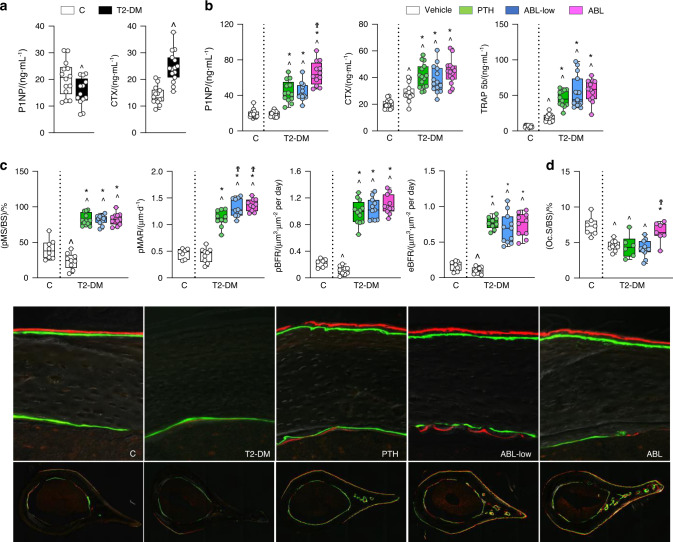
PTH and ABL reversed the impaired osteoblast function in diabetes, but ABL was more potent. Serum P1NP and CTX measure **a** before (t3) and **b** P1NP, CTX, TRAP5b measure after (t4) treatment with PTH or ABL. **c** Dynamic bone histomorphometric analysis of periosteal mineralized surface/bone surface (MS/BS) and mineral apposition rate (MAR) and periosteal and endosteal bone formation rate (BFR) of the cross-section of the femoral mid-diaphysis and representative images. **d** Surface covered by osteoclasts (OcS/BS). *n* = 12–15 mice per group. Data are presented as box & whisker plot where each dot represents a mouse. ^*P* < 0.05 versus C mice by unpaired and two-tailed Student’s t-test (**a**) or one-w**a**y ANOVA with post hoc Dunnet’s correction (**b**–**d**); *P < 0.05 versus T2-DM mice treated with vehicle and ^Ϯ^*P* < 0.05 versus T2-DM mice treated with PTH by one-way ANOVA with post hoc Tukey’s correction

Circulating levels of the bone formation marker P1NP were reduced in T2-DM mice compared to C before treatment at t3 (Fig. 3a); and PTH and ABL-low were equally potent in increasing P1NP (130% vs C and 129% vs T2-DM; *P* < 0.05) whereas the equimolar dose of ABL increased it further (240% vs C and 50% vs PTH/ABL-low; *P* < 0.05) (Fig. 3b). In cortical bone, bone formation rate (BFR) was reduced in T2-DM mice compared to C in both the periosteal and endocortical surfaces, resulting from a reduction in MS/BS (BFR −50% and −35%, and MS/BS −40% and −24%, respectively; *P* < 0.05) (Fig. 3c and Fig. S2b). ABL and PTH were equally potent in increasing all indexes of bone formation on both surfaces in T2-DM, far beyond the C non-diabetic mice. On the periosteal surface, PTH/ABL increased MS/BS by 200% and BFR by 700% vs vehicle-treated T2-DM mice. In addition, periosteal MAR was increased by PTH (160% vs vehicle-T2-DM mice; *P* < 0.05) and further enhanced by either dose of ABL (20% vs PTH; *P* < 0.05). The treatments increased to the same extent MAR, MS/BS, and BFR on the endocortical and cancellous bone surfaces (Fig. S2b, c).

**Fig. 4:**
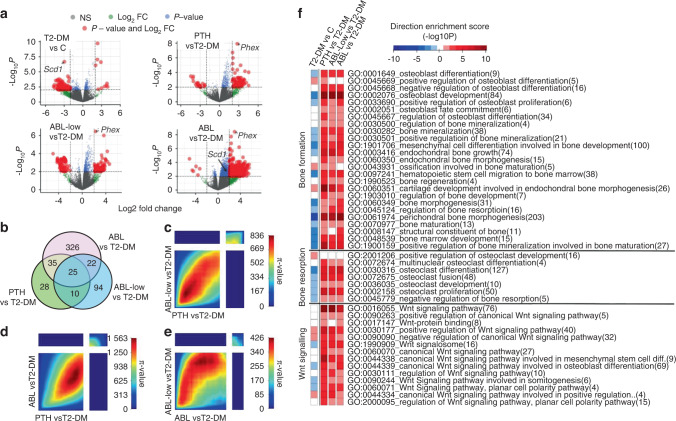
PTH and ABL generated shared and unique molecular signatures in bone and corrected the dysregulated bone remodeling and Wnt signaling transcriptome induced by T2-DM. **a** Volcano plots illustrate numbers of significantly downregulated or upregulated DEGs for each comparison. **b** Venn diagram depicts the number of unique and shared DEGs in L4 bones. **c**–**e** The π-value heatmaps resulting from Rank-Rank Hypergeometric Overlap (RRHO) analysis show high concordance in the gene expression signatures of PTH and ABL in the context of T2-DM. **f** A directional gene set enrichment analysis of the gene ontology (GO) terms in the DEGs was performed using the PIANO package. The heat map shows the value of the enrichment score (-log10(enrichment *P*-value)). The GO terms are color-coded to illustrate the direction of the gene expression changes in the majority of genes included in the respective GO term, i.e., red for upregulation and blue for downregulation

Regarding resorption, T2-DM mice exhibited high levels of circulating CTX before treatment (Fig. 3a), which remained elevated in vehicle-treated T2-DM mice and were further increased by PTH and either dose of ABL (100% vs C and 40% vs T2-DM; *P* < 0.05). Similarly, T2-DM mice exhibited increased circulating levels of TRAP 5b, a marker of osteoclast number, and PTH/ABL further increased it (700% vs C and vs 200% vs T2-DM; *P* < 0.05). In contrast, osteoclasts were decreased in bone sections from T2-DM mice compared to non-diabetic mice at the end of the study. And, although PTH or ABL-low did not change osteoclast number in T2-DM mice, the high dose of ABL increased osteoclast surface (Fig. 3d and Fig. S2d). These findings are consistent with a decrease by diabetes and conversely an increase by PTH/ABL in processes associated with resorption detected by gene ontology (GO) analysis (Fig. 4f). Overall, these findings at the tissue level support the notion that established diabetes is a low bone remodeling disease; and that PTH/ABL reverse it towards a high bone remodeling condition with bone gain.

### PTH and ABL corrected the diabetic transcriptome signature of low bone remodeling and decreased Wnt signaling

Bone transcriptome analysis, based on a cutoff of log2 fold change (FC) > 2 or < −2, *P* < 0.01, revealed a high number of genes downregulated by T2-DM and, conversely, a high number of genes upregulated by treatments, in particular by ABL shown in the volcano plots (Fig. 4a). From the 112 differentially expressed genes (DEG) in diabetic bones, 102 were downregulated and 10 up-regulated. Treatment of T2-DM mice with the PTH1R ligands change the bone transcriptome. A limitation of the transcriptome signature is that hematopoietic cells were included in the RNA-seq analysis, potentially masking some of the effects of PTH/ABL on mesenchymal cells. PTH increased the expression of 78 genes and decreased the expression of 20 genes; ABL-low increased the expression of 72 genes and decreased the expression of 79 genes; and ABL increased the expression of 402 genes and decreased the expression of 6 genes (Fig. 4a). Four of the genes upregulated in diabetic bone have been previously associated with glucose homeostasis, lipid metabolism, or pancreatic β cell function (Table 1).^11–13^ Among the genes downregulated by T2-DM, Stearoyl–coenzyme A desaturase-1 (*Scd1*) was the most significantly downregulated (−2.9 fold), and it was conversely significantly upregulated only by ABL ( + 1.4 fold) (Fig. 4a and Table 1). *Scd1* has previously shown to stimulate osteogenic differentiation,^14^ and to be downregulated in bone marrow stromal cells from T2-DM patients.^15^ Regulator of G protein signaling like 1 (Rgsl1) and Forkhead box protein F1 (Foxf1) were also downregulated in T2-DM bones and were upregulated by both PTH and ABL. Future studies will be required to establish the role of these conversely regulated genes in diabetes-induced bone disease and/or PTH/ABL action in bone.

**Table 1:** Differentially expressed genes in T2-DM mice treated with PTH or ABL (Fig. 4a)

GENE	T2-DM + veh vs control		T2-DM + PTH vs T2-DM		T2-DM + ABL-low vs T2-DM		T2-DM + ABL vs T2-DM	
**UP-REGULATED**	**logFC**	**log10 (*P*.val)**	**logFC**	**log10 (*P*.val)**	**logFC**	**log10 (*P*.val)**	**logFC**	**log10 (*P*.val)**
Angptl4	2.40	9.68	0.37	1.00	0.60	1.94	0.43	1.22
Acot1	2.35	7.00	0.32	0.58	0.53	1.02	0.43	0.77
BC048679	2.92	6.57	−0.41	0.50	0.03	0.03	−0.67	0.97
Fbp2	2.07	6.05	0.37	0.62	0.58	1.14	0.34	0.56
Tmco5b	3.89	3.03	−3.37	2.49	−2.32	1.49	−1.83	1.07
Kcnmb3	3.80	2.08	−0.38	0.18	0.54	0.27	−0.60	0.31
**DOWN-REGULATED**	**logFC**	**log10 (*P*.val)**	**logFC**	**log10 (*P*.val)**	**logFC**	**log10 (*P*.val)**	**logFC**	**log10 (*P*.val)**
Scd1	−2.94	6.66	0.35	0.41	0.56	0.76	1.43	2.80
Timd4	−2.39	4.25	0.85	1.04	−0.12	0.10	0.80	0.96
Cyp2e1	−2.17	3.92	−0.57	0.63	0.50	0.53	0.19	0.16
Reg4	−3.50	3.57	3.95	4.16	0.42	0.22	3.14	3.10
Rgsl1	−4.31	3.54	3.72	2.92	3.48	2.67	4.82	4.07
Vmn1r21	−4.10	3.53	1.28	0.72	2.01	1.33	4.03	3.46
Scgb3a1	−2.26	3.38	2.77	4.35	3.66	6.01	3.77	6.20
Hsf3	−3.47	3.26	2.97	2.65	1.28	0.82	3.80	3.67
Tescl	−3.74	3.25	1.54	1.18	3.40	3.50	3.47	3.58
BC061237	−3.22	3.08	3.11	2.68	1.78	1.23	3.06	2.62
Foxf1	−3.79	2.95	3.03	2.94	2.38	2.11	3.54	3.60
Gpr101	−3.45	2.93	2.15	1.94	1.99	1.75	3.08	3.18

Consistent with the role of the PTH1R in osteocytes,^16,17^ all treatments increased the expression of several osteocytic genes, being phosphate regulating endopeptidase X-linked (*Phex*) the most significantly upregulated (PTH + 2.3 fold; ABL-Low + 2.3; ABL + 3.0) (Fig. 4a). In addition, common and unique set of genes were transcriptionally regulated by PTH vs ABL. Twenty-five genes were transcriptionally regulated by all treatments, while PTH exclusively regulated 28 genes, ABL-low 94 genes, and ABL 326 genes (Fig. 4b). These findings demonstrate that ABL induced the most significant modification of the bone gene transcriptome compared to PTH in the frame of diabetes.

To further evaluate the transcriptional responses of the ligands of the PTH1R, we compared the gene expression signatures of PTH, ABL-low and ABL in the context of T2-DM using the threshold-free approach Rank-Rank Hypergeometric Overlap (RRHO).^18^ The RRHO analysis detected no discordant transcriptional patterns among treatments (top-left and bottom-right quadrants, Fig. 4c–e). In contrast, it detected high concordance in the transcriptionally up-regulated and down-regulated genes (bottom-left and top-right quadrants, respectively) by PTH and ABL, as analyzed by pairwise comparisons. Moreover, the bigger size of the bottom-left quadrant compared to the top-right quadrant indicates that the majority of the genes regulated by the treatments were up-regulated.

GO enrichment mapping identified biological processes affected by T2-DM and/or the treatments with PTH1R ligands. Among them, three biological processes were selected: bone formation, bone resorption, and Wnt signaling (Fig. 4f). The GO term groups of genes related to these three processes were overall decreased by T2-DM and, conversely, increased by the different treatments to the same extent, as shown in the heat maps. The gene signatures of bone formation and resorption are consistent with the tissue-level responses to diabetes and the treatments with the PTH1R ligands. Specifically, bone formation rate (BFR) and osteoclast number are decreased in diabetic bone and increased by administration of PTH/ABL.

### Scl-Ab antibody restored the bone lost with diabetes by increasing bone formation and reducing osteoclasts

**Fig. 5:**
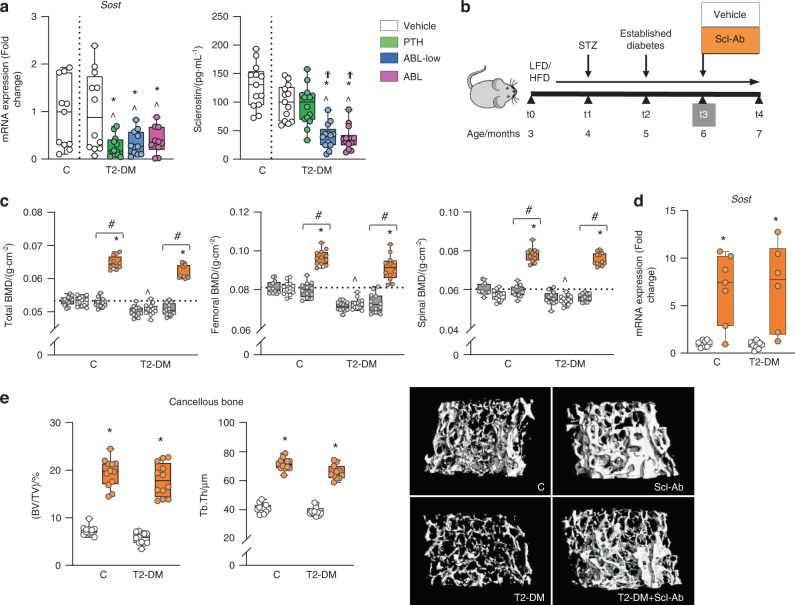
PTH and ABL decreased Sost expression in T2-DM bone, and the anti-sclerostin antibody restored and further increased BMD and cancellous bone in T2-DM. **a** Expression of the osteocytic marker *Sost* in bone and Sclerostin levels in serum of DM mice after treatment with PTH or ABL (at t4). **b** Study design: Male C57BL/6 DM mice as well as C mice were administered with vehicle or Scl-Ab once a week for 4 weeks. **c** Total, femoral and spinal BMD before treatment (at t3, grey bars) and after treatment (at t4) with vehicle (white bars) or Scl-Ab (orange bars). **d** Expression of *Sost* in bones of C and DM mice after (at t4) treatment with vehicle or Scl-Ab. **e** Micro-CT analysis of femur cancellous bone: BV/TV and trabecular thickness (Tb.Th), after treatment with vehicle or Scl-Ab and representative micro-CT images. *n* = 10-12 mice/group. Data are presented as box & whisker plots where each dot represents a mouse. ^*P* < 0.05 versus C mice by one-way ANOVA for (**a**) or two-w**a**y ANOVA with post hoc Dunnet’s correction for (**c**). For (**a**), *P < 0.05 versus T2-DM mice treated with vehicle and ^Ϯ^*P* < 0.05 versus T2-DM mice treated with PTH by one-way ANOVA with post hoc Tukey’s correction. For (**c**–**e**), *P < 0.05 versus respe**c**tive vehicle-treated mice by two-way ANOVA with post hoc Bonferroni’s correction. ^#^*P* < 0.05 versus respective mice at t3 (grey bars) by one-way ANOVA with post hoc Tukey’s correction

The GO enrichment mapping also identified Wnt signaling, a pathway that pays a central role in osteogenesis and the control of bone mass, as a biological process downregulated by T2-DM and, conversely, upregulated by PTH/ABL (Fig. 4f). Earlier findings showed that bones from T1-DM mice exhibited increased expression of Sost, the osteocyte-derived Wnt signaling antagonist and inhibitor of bone formation.^19^ In contrast, in the current study T2-DM mice exhibited similar Sost expression in bone or sclerostin levels in serum compared to C mice (Fig. 5a). Nevertheless, PTH and both doses of ABL decreased bone mRNA Sost expression and ABL also reduced circulating sclerostin protein levels (Fig. 5a), suggesting a potential role of Sost/sclerostin downregulation in the anabolic function of the PTH1R ligands in the context of diabetes.

We next therefore investigated whether sclerostin inhibition per se was sufficient to reverse the bone deterioration induced by T2-DM. The humanized monoclonal anti-sclerostin antibody (Scl-Ab)^20^ was administered to non-diabetic or T2-DM mice for 4 weeks (Fig. 5b and Fig. S3a, b). Scl-Ab increased BMD at all sites in C mice; and in T2-DM mice, Scl-Ab not only restored the bone lost with diabetes but further increased BMD over the value of C mice (Fig. 5c). Scl-Ab treatment increased the expression of Sost in C and T2-DM mice (Fig. 5d). Blood glucose, body weight, and fat mass remained elevated in T2-DM mice treated with Scl-Ab (Fig. S3d, e).

**Fig. 6:**
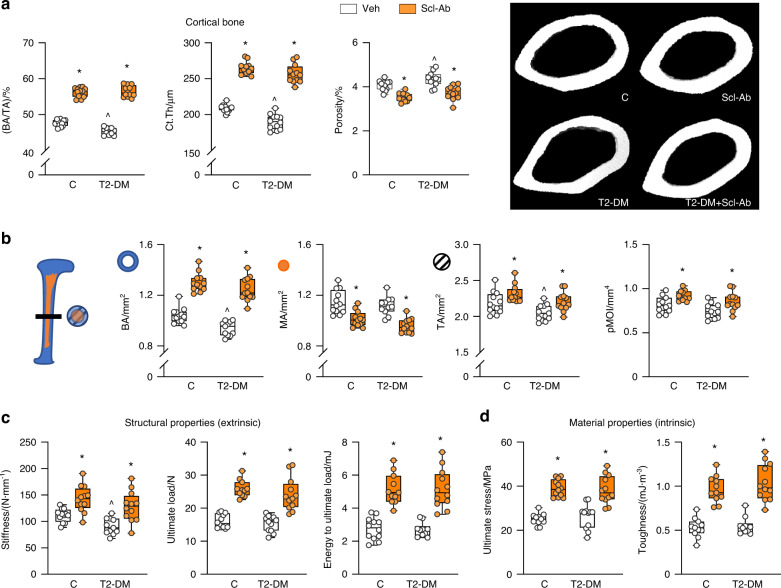
Scl-Ab restored and further increased cortical bone volume and improved microarchitecture and extrinsic and intrinsic bone properties in both control and diabetic mice. Micro-CT analysis of femur bone microarchitecture and 3-point bending analysis of bone strength after (t4) treatment with Scl-Ab. **a** Cortical bone area/tissue area (BA/TA), thickness (Ct.Th) and porosity and representative images. **b** Bone area (BA), medullary area (MA), total area (TA) and polar moment of inertia (pMOI). **c** Bone biomechanical properties (stiffness, ultimate load, and energy to ultimate load) and **d** bone material properties (ultimate stress and toughness). For all analyses, *n* = 10–12 mice per group. Data are presented as box & whisker plot where each dot represents a mouse. ^*P* < 0.05 versus control LFD mice and *P < 0.05 versus respective vehicle-treated mice by two-way ANOVA with post hoc Bonferroni’s correction for multiple comparisons

Scl-Ab reversed the T2-DM-induced architectural changes in trabecular and cortical bone of the femur as well as improved bone architecture in C mice. In trabecular bone, Scl-Ab corrected and further increased BV/TV and connectivity density, increased trabecular thickness, and number and reduced separation, SMI, and material density (Fig. 5e and Table S3). In cortical bone, Scl-Ab increased BA/TA, and cortical thickness by both increasing tissue area and reducing marrow area (Fig. 6a and Table S3). These effects resulted in structural changes leading to increased pMOI. Moreover, Scl-Ab reduced cortical porosity in C mice and corrected the increased porosity in diabetic bone (Fig. 6a, b and Table S3). Scl-Ab also corrected the structural changes induced by diabetes and improved both structural and material properties of bone in both T2-DM and C mice (Fig. 6c, d and Table S3). The effect of diabetes was more pronounced in the vertebra, as L6 BV/TV and trabecular number were reduced in T2-DM mice (Table S4). Nevertheless, Scl-Ab efficiently corrected vertebral trabecular bone deterioration as in the femur.

**Fig. 7:**
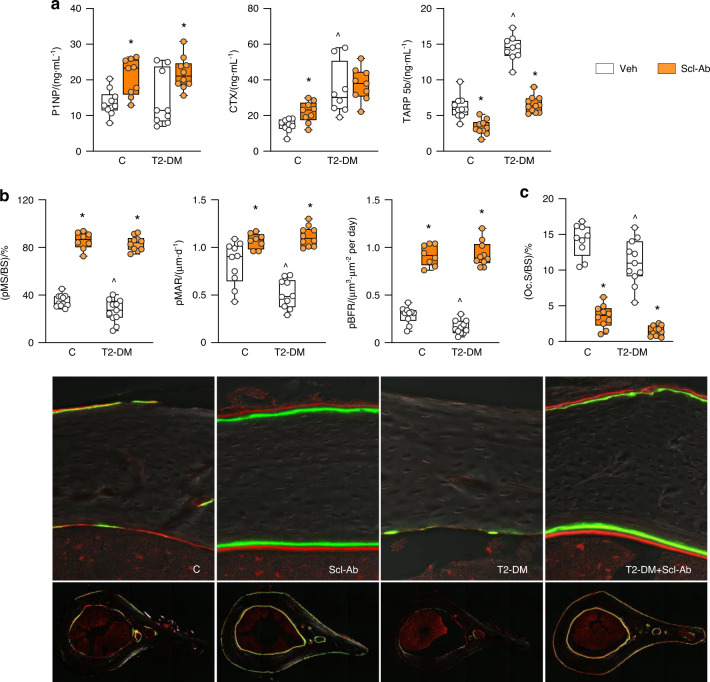
Scl-Ab increased bone formation, inhibited bone resorption and reversed the impaired osteoblast function in diabetic mice. **a** P1NP, CTX, TRAP 5b measure after treatment with Scl-Ab in C and DM mice. **b** Dynamic bone histomorphometric analysis of mineralized surface/bone surface (MS/BS), mineral apposition rate (MAR) and bone formation rate (BFR) at the periosteal surface of the cross-section of the femoral mid-diaphysis after treatment with Scl-Ab in control and DM mice and representative images. **c** Surface covered by osteoclasts (OcS/BS). For all analyses, *n* = 10–12 mice per group. Data are presented as box & whisker plot where each dot represents a mouse. ^*P* < 0.05 versus control LFD mice and *P < 0.05 versus respective vehicle-treated mice by two-way ANOVA with post hoc Bonferroni’s correction for multiple comparisons

As shown in the PTH/ABL study, P1NP was decreased in T2-DM mice before treatment (at t3, Fig. S4a), but was similar to C levels at the end of the experiment (at t4, Fig. 7a); and treatment with Scl-Ab increased P1NP in both T2-DM (52%, *P* < 0.05) and C mice. Moreover, T2-DM mice exhibited the expected increase in circulating levels of TRAP5b (200% vs C, *P* < 0.05) and CTX (115% vs C, *P* < 0.05), and the Scl-Ab decreased TRAP5b levels but not CTX.

Furthermore, Scl-Ab increased BFR on both periosteal (Fig. 7b) and endocortical (Fig. S4b) bone surfaces of femoral mid-diaphysis. In contrast with the effects of the PTH1R ligands, treatment with the Scl-Ab markedly reduced osteoclast number/surface on bone (Fig. 7c and Fig. S4c).

## Discussion

The bone fragility syndrome associated with diabetes causes substantial morbidity, decreases quality of life, and diminishes life expectancy of patients, with the associated high health-care costs. Yet, diabetes-induced bone disease is under-recognized, under-treated, and of unclear underlying mechanisms. Furthermore, anti-resorptive agents stop the bone loss but do not rebuild bone nor restore the deteriorated architecture of the diabetic skeleton. Herein, we report the diabetic bone signature characterized by low bone remodeling and decreased Wnt signaling and demonstrate that the three FDA-approved bone anabolic agents correct it at the tissue, cellular and transcriptome levels. The bone protective effects of the anabolic therapies were independent of the diabetic status, as the loss of mineral, the damaged architecture, and the low activity of the bone-forming cells were corrected despite the presence of overt hyperglycemia. Our pre-clinical mechanistic evidence suggests the need for revisiting the current treatment recommendations for bone fragility in diabetes and highlights the potential applicability of bone anabolic regimens to restore skeletal strength regardless of the presence or not of active diabetic disease.

Our non-genetic model of T2-DM uses skeletally mature mice, mimics the adult onset of diabetes, and closely mirrors the course of the bone disease induced by diabetes observed in humans. Hyperglycemia in T2-DM can result from insulin resistance and/or insulin deficiency.^21^ As shown earlier by Eckhardt et al.,^22^ the HFD/STZ mouse model is characterized by persistent hyperglycemia, body fat accumulation, dysfunctional insulin secretion, and loss of pancreatic β cells, but not high insulinemia. Moreover, it is vastly documented that HFD alone is not sufficient to achieve hyperglycemia and that combination of HFD with STZ is required to mimic T2-DM in wild-type rodents.^23^ Therefore, despite the limitations of every model, the HFD/STZ combination is currently the most reliable model of T2-DM in rodents.

The initial high bone resorption leading to bone loss in our model is evidenced by elevated levels of circulating markers of resorption, which remain high throughout. However, at the end of the study, osteoclasts on bone are low and the expression of resorption-associated genes detected by transcriptome analysis is reduced. This temporal change indicates a transition from high resorption to the low resorption condition that characterizes the bone disease in humans with established diabetes. The apparent discrepancy between serum markers and resorption at the tissue level at the end of the study might be explained by the dynamics of the protein marker turnover in the circulation. On the other hand, bone formation is suppressed throughout the entire disease progression, as evidenced by low serum bone formation markers and at the tissue level by the decreased bone formation rate and gene ontology enrichment mapping. Thus, similar to humans, established diabetes in our model is a low bone remodeling disease.^24–26^ Further, the model reproduces the architectural characteristics of the disease in humans including increased porosity of cortical bone, thinning of cortices and trabeculae, and decreased vertebral cross-sectional area.^27^ These features are accompanied by a decrease in stiffness, a structural/extrinsic property and key component of overall bone strength.

Based on the underlying mechanisms of the skeletal disease in diabetes, bone anabolic agents capable of increasing bone formation and repairing the lost bone should be preferred compared to antiresorptive agents. We indeed demonstrate in the current study the effectiveness of two bone anabolic pathways: activation of the PTH1R and neutralization of the osteocyte-derived inhibitor of bone formation sclerostin. Our findings are consistent with post hoc analysis of large clinical osteoporosis trials demonstrating that teriparatide, ABL, or romosozumab increased BMD and trabecular bone score and reduced non-vertebral fracture incidence similarly in diabetic and non-diabetic patients.^28–31^

We compared and contrasted for the first time the efficacy of two anabolic ligands of the PTH1R: PTH(1-34) and ABL on restoring bone health in established diabetes, and found that ABL is more potent and provides better bone geometry benefits compared to PTH. Thus, half a dose of ABL was sufficient to restore bone mass, increase bone formation, and correct architectural deterioration and bone strength in diabetic mice to a similar extent than PTH, and the equimolar dose of ABL did not provide additional advantages compared to the half dose. In addition, although both PTH and ABL restored cortical bone thickness, they acted on different surfaces, as PTH reduced marrow area whereas ABL increased tissue area, with the consequent change in bone geometry and polar moment of inertia, leading to increased strength with ABL. Overall, PTH and ABL increased to a similar extent BFR on all bone surfaces. Thus, the higher levels of the bone formation marker P1NP in response to the high dose of ABL a priori cannot be attributed to overall changes in BFR at the tissue level. However, ABL compared to PTH induced a greater increase in MAR, one of the components of BFR, on the periosteal surface of cortical bone, leading to the changes in bone geometry. Therefore, higher P1NP might reflect this differential effect of ABL vs PTH on cortical bone surfaces.

The greater gain in bone induced by ABL compared to PTH in the clinic was originally attributed to lack of increase in bone resorption by ABL shown in preclinical reports.^32,33^ Also, both peptides increased formation markers to the same extent, but ABL induced less prominent increases in resorption compared to PTH in clinical studies.^34^ In contrast, other studies showed that similar doses of PTH and ABL increase resorption markers and osteoclasts to the same level.^35,36^ Our study in the context of diabetes demonstrates that PTH and ABL increased resorption markers at comparable levels when tested at equimolar doses, and that even half dose of ABL induced a similar response. Furthermore, the high dose of ABL increased osteoclasts on bone. Thus, the bone gain induced by activation of the PTH1R with either ligand in diabetes is characterized by increased bone formation in the presence of sustained bone resorption. On the other hand, bone restoration and the increased bone formation in diabetes by the anti-sclerostin antibody are accompanied by decreased resorption, consistent with the profile of Scl-Ab in preclinical^33^ and clinical studies with osteoporotic patients.^37,38^

In closing, our findings demonstrate the efficacy of increasing bone formation to restore the damaging effects of diabetes on bone (Graphical Abstract). The dysregulated bone remodeling and deteriorated microarchitecture of diabetic bone were corrected by all three FDA-approved agents with bone anabolic properties, and simultaneous inhibition of resorption with the dual action agent Scl-Ab led to superior bone gain and strength compared to the purely anabolic ligands of the PTH1R. This evidence opens new avenues to treat the skeletal fragility in diabetic patients.

## Materials and methods

### Animals

All animal procedures were approved by the Institutional Animal Care and Use Committee at University of Arkansas, and animal care was carried out in accordance with institutional guidelines.

Twelve-week-old male C57BL/6 J mice (The Jackson Laboratory, Bar Harbor, ME) were housed 5 mice/cage, received water *ad-libitum* and were exposed to a 12 h light/dark cycle. Mice were fed a low-fat diet (LFD, 10 kcal% fat, D12450J) or high-fat diet (HFD, 60 kcal% fat, D12492) throughout the experiment. At t1, type 2 diabetes (T2-DM) was induced in HFD-fed mice by 5 daily injections of streptozotocin (STZ, 45 mg·kg^−1^ i.p. in 50 mmol·L^−1^ citrate buffer, pH 4.5) while control LFD-fed mice received citrate buffer (C), adapted from.^22^ At t2, 4 weeks after initiating the STZ injections, DM was confirmed in fasted animals by blood glucose values > 250 mg·dL^−1^.^19^ After 4 weeks at t3, C and T2-DM mice were stratified based on BMD, glucose levels, and weight into treatment groups. Then, mice were injected s.c. 7 days a week with vehicle (0.9% saline, 0.01 mmol·L^−1^ β-mercaptoethanol, 0.1 mmol·L^−1^ acetic acid), PTH (100 μg·kg^−1^ per day, Bachem, Torrance, CA), or ABL (47.5 and 95 μg·kg^−1^ per day, Radius Pharmaceutical, Boston, MA); or i.p. once a week with vehicle (saline) or human Scl-Ab (100 mg·kg^−1^ per week romosozumab, ^©^Amgen Inc). The dose of PTH (100 μg·kg^−1^ per day or 24 pmol·g^−1^ per day) was chosen to attain optimal responses based on earlier studies.^39–42^ The dose of ABL (95 μg·kg^−1^ per day or 24 pmol·g^−1^ per day) was chosen to allow a direct mole to mole comparison with PTH. Half dose of ABL (47.5 μg·kg^−1^ per day or 12 pmol·g^−1^ per day) was also used based on our earlier study with the PTHrP (1-37) and diabetic mice^19^ and studies using ABL in ovariectomized rats.^32,43^ The dose of Scl-Ab (100 mg·kg^−1^ per week) was chosen to maximize the bone anabolic response and prevent immunogenicity against the human monoclonal antibody.^44^

Endpoint measurements were performed 28 days after the first injection of the anabolic agents (t4), followed by euthanasia and tissue harvesting (Figs. 1a and 5b).

### Analysis of skeletal phenotypes

#### Bone mineral density measurements

Longitudinal study was performed at t0, t1, t2, t3, and t4 by dual-energy x-ray absorptiometry (DEXA) using a PIXImus densitometer (G.E. Medical Systems,Lunar Division, Madison, WI). BMD measurements included total BMD (excluding the head and tail), L1–L6 vertebra (spinal BMD), and entire femur (femoral BMD).^19^ Mice were stratified to the experimental groups based on BMD, glucose levels and body weight measured at t0, t1, t2, and t3.

#### Bone microarchitecture analysis

For microcomputed tomography (μCT) analysis, bones were dissected, cleaned of soft tissue, and stored in saline at −20 °C. The femurs and L_6_ vertebrae were scanned in a μCT scanner. Bones from the PTH/ABL experiment were analyzed using a μCT40 (E = 55 kVp, I = 145 uA, integration time = 200 ms, Scanco Medical, Switzerland) at an isotropic voxel size of 10 µm. Bones from the Scl-Ab experiment were analyzed using a vivaCT80 (E = 70 kVp, I = 114 uA, integration time = 200 ms, Scanco Medical, Switzerland) at an isotropic voxel size of 10.4 µm. For the trabecular analysis a Gaussian filter (sigma = 1.2, support = 1) was applied and a threshold of 285 mg·cm^−3^ was used. Femoral distal cancellous bone measurements were analyzed beginning 10 slices away from the growth plate to avoid the primary spongiosa for 151 slices.

L_6_ cancellous bone measurement were performed using a VOI spanning from the upper to the lower growth plate excluding primary spongiosa. Cortical bone was measured at a threshold of 260 mg·cm^−3^. Femoral mid-diaphysis cortical analysis was performed for 20 slices region located at the calculated femoral midpoint. L6 cortical bone analysis was performed starting 10 slices away (towards caudal growth plate) from where the first spinous process attaches to the vertebral bod for 10 slices.^45^ All nomenclature, symbols, and units adhered to guidelines in the literature.^46^

#### Bone histomorphometric analysis

To measure the dynamic histomorphometric indexes mineralizing surface to bone surface (MS/BS), mineral apposition rate (MAR), and bone formation rate normalized to bone surface (BFR/BS), bone multicolor fluorochrome labeling^47^ was performed by i.p. injections with calcein green (G, 30 mg·kg^−1^ bw) and alizarin red (A, 50 mg·kg^−1^ bw) in the order of G-A at days 18 and 25 (initiation of PTH/ABL, Scl-ab or VEH on day 0), followed by euthanasia at day 28. Left femurs were fixed in 10% buffered formalin, cut in half at the midshaft, then embedded undecalcified in methyl methacrylate. Thick cross-sections at the mid-diaphysis were prepared using a diamond-embedded wire saw (Histosaw, Delaware Diamond Knives, Wilmington, DE, USA) and grounded to a final thickness of 30–40 µm for periosteal and endosteal bone formation measurements.^48^

For static bone histomorphometric analysis, longitudinal sections of the distal femurs were stained for TRAPase and counterstained with Toluidine blue as previously published.^49^ TRAPase^+^ cells with three or more nuclei attached to the femoral cancellous bone region (starting 350 μm from the distal growth plate and ending 1 750 μm proximal to the distal growth plate) were quantified as osteoclast. Histomorphometric analysis was performed using OsteoMeasure High Resolution Digital Video System (OsteoMetrics, Decatur, GA) interfaced to an Olympus BX51 fluorescence microscope (Olympus America Inc., Center Valley, PA).^48^ Terminology and units are those recommended by the Histomorphometry Nomenclature Committee of the ASBMR.^50^ Analyses were performed in a blinded fashion.

### Bone turnover markers

Blood was collected at t0, t1, t2, and t3 from the facial vein of 3 h fasted mice. Procollagen type 1 N-terminal propeptide (P1NP), C‐telopeptide fragments of type I collagen (CTX), tartrate-resistant acid phosphatase form 5b (TRAP 5b) (RatLaps, Immunodiagnostic Systems Inc., Fountain Hills, AZ, USA), and SOST/Sclerostin (R&D Systems, Minneapolis, MN, USA) were measured following the manufacturer’s instructions.

### Biomechanical testing

Following microCT scanning, femurs and L6s were subjected to mechanical test to assess the mechanical and material properties. The femurs were subjected to 3-point bending on an Instron model 5542 with a ramp rate of 1 mm per minute and an 8.1 mm support span (L) until failure. The analysis was run using Bluehill2 software ver. 2.35. The vertebrae were subjected to a vertebral compression test on the Instron 5542 with a ramp of 0.5 mm per minute.^51^ Structural or extrinsic properties (energy to ultimate load, ultimate load, and stiffness) of the femur and L6 were derived from the load/displacement curves obtained during the three-point bending tests. Cross-sectional polar moment of inertia (I) and anterior-posterior diameter (d) were determined by µCT and were used to calculate material or intrinsic properties including ultimate stress (FLd/8I, where F = ultimate load, L = span length, d = anterior-posterior diameter, and I = cross-sectional moment of inertia), modulus (SL^3^/48I, where S = stiffness) and toughness (0.75 ∗ AUC d^2^ /LI, where AUC = area under curve [i.e., energy to ultimate load] and b = diameter).

### RNA extraction and quantitative PCR (qPCR)

Dissected murine bone tissues were snap-frozen in liquid nitrogen and stored at −80 °C. Lumbar vertebrae, tibial diaphysis and calvarial bones were homogenized in Trizol Reagent (Life Technologies # 15596018), and RNA was isolated using a Direct-zol RNA Miniprep Plus Kit (Zymo Research, # R2072) according to the manufacturer’s instructions. RNA concentrations and the 260/280 ratios were determined using a Nanodrop instrument (Thermo Fisher Scientific). 1 µg of RNA from each tissue was used to synthesize cDNA using a High-Capacity cDNA Reverse Transcription Kit (Applied Biosystems #4368814) according to the manufacturer’s instructions.

Gene expression was quantified by qPCR as described earlier^52^ using sets of primers and probes designed using the Assay Design Center (Roche Applied Science, Indianapolis, IN, USA) or commercially available, carried out in an ABI PRISM 7500 system (Applied Biosystems, Foster City, CA, USA). Relative mRNA expression was normalized to the housekeeping glyceraldehyde-3-phosphate dehydrogenase (GAPDH) using the *Δ*Ct method. Ratios between genes of interest and housekeeping gene are expressed as fold change compared with mice receiving placebo.

### RNA-seq analysis

For each experimental group, *n* = 5 high-quality RNA samples from the 4th lumbar vertebrae were subjected to RNA-seq analysis on an Agilent platform (Agilent Technologies). The RNA samples that have RIN number > 7 were used for further step. Preparation of RNA sequencing library (mRNA) and transcriptome sequencing was conducted by Novogene Co., LTD (Beijing, China). The libraries were sequenced in Illumina NovaSeq platforms to generated paired-end 150 bp read length.

### Data acquisition and analysis

The RNA-seq analysis was performed follow our bioinformatic pipeline.^53^ FastQ files were align to the reference genome of Mus musculus version GRCm39 with gene annotation version 105, downloaded form Ensemble genome database, using STAR software version2.7.9a.^54^ The gene count table were generated using BEDTools version 2^55^ and imported to R suite software version 4.02. for further analysis. The count data was normalized using voom method^56^ with quantitative quality weights.^57^ Limma package^58^ was employ for differential gene expression analysis using the moderate Student’s t-test to compare the different groups with the control group at each time point, and the *P*-values were further adjusted for multiple testing using the Benjamini-Hochberg method. Changes in expression induced by the PTH and ABL were calculated by dividing the log2 expression value of each individual adjuvant-treated group by the log2 expression value of the T2-DM group.

The statistical π-value^59^ was calculated by multiplying the -log10Pvalues and log2 fold changes of individual genes. The π-values were used to assess the concordant and discordant gene expression patterns induced by the treatments in the frame of T2-DM using Rank-Rank Hypergeometric Overlap (RRHO) method (RHHO2 package).^18^ Results were plotted as the spited heatmap.

A directional gene set gene ontology (GO) enrichment analysis was performed using the PIANO package.^60^ GO relationships were retrieved from Ensemble database as was used as a scaffold to integrate the differentiation gene expression results. The enrichment results were plotted as a heatmap of the enrichment score [−log10 (enrichment *P*-value)]. https://dataview.ncbi.nlm.nih.gov/object/PRJNA895324?reviewer=8m72hr7tk1mkjg3h012pto6sq6

### Statistical analysis

Statistical analysis was performed using GraphPad Prism 9.3.0 (GraphPad Software, Inc., CA, US). For each measure of interest, Levene’s test was employed to investigate homogeneity of variance between groups. In case of equal variances, ANOVA methods were employed. When homogeneity of variance assumptions were not satisfied, non-parametric methods (Kruskal-Wallis test) were utilized. When overall effects were significant (*P* < 0.05), two-sided post- hoc pairwise comparisons were performed (parametric: Tukey, Bonferroni or Dunnett test, as appropriate; non-parametric: Wilcoxon rank sum test). Statistical details of each experiment (test used and value of n) can be found in Figure and Figure Legend sections. Data are presented as boxplots overlayed with dot plots, where each dot represent a mouse.

## Supplementary information

Supplemental tables

Supplemental Figures

## Data Availability

All data associated with this study are available upon request.
