# Author Correction: Reversal of the diabetic bone signature with anabolic therapies in mice

**DOI:** 10.1038/s41413-023-00266-9

**Published:** 2023-05-06

**Authors:** Silvia Marino, Nisreen Akel, Shenyang Li, Meloney Cregor, Meghan Jones, Betiana Perez, Gaston Troncoso, Jomeeka Meeks, Scott Stuart, Amy Y. Sato, Intawat Nookaew, Teresita Bellido

**Affiliations:** 1 Department of Physiology and Cell Biology, University of Arkansas for Medical Sciences, Little Rock, AR USA; 2 Central Arkansas Veterans Healthcare System, John L. McClellan Little Rock, Little Rock, AR USA; 3 Department of Biostatistics, University of Arkansas for Medical Sciences, Little Rock, AR USA; 4 Department of Biomedical Informatics, University of Arkansas for Medical Sciences, Little Rock, AR USA; 5 Winthrop P. Rockefeller Cancer Institute, University of Arkansas for Medical Sciences, Little Rock, AR USA

**Keywords:** Bone quality and biomechanics, Pathogenesis

Correction to: *Bone Research* 10.1038/s41413-023-00261-0, published online 19 April 2023

Following publication of this article,^1^ it is noticed that a sentence “and National Institute of General Medical Sciences of the National Institutes of Health P20GM125503 to IN” should be added to the Acknowledgement section.

The correction Acknowledgement should read:

Authors acknowledge Jeffrey A Kamykowski from the UAMS Digital Microscopy Core for assistance with image acquisition, and Stuart B. Berryhill, Julie Crawford, Richard D. Peek from the UAMS Bone Biomechanics, Histology and Imaging Core of the Center for Musculoskeletal Disease Research (Center of Biomedical Research Excellence COBRE), and Jesus Delgado-Calle for critical reading of the manuscript. Abaloparatide (ABL) was provided by Gary Hattersley and Beate Lanske from Radius Health, Inc.

This research was supported by the Veterans Administration I01 BX002104 and IK6BX004596 to T.B.; R01-AR059357 to T.B.; UAMS College of Medicine Sturgis Endowment Grant to T.B.; ASH scholar award to S.M.; National Center for Advancing Translational Sciences of the National Institutes of Health KL2TR003108 and UL1TR003107 to A.Y.S.; and National Institute of General Medical Sciences of the National Institutes of Health P20GM125503 to I.N. The contents do not represent the views of the U.S. Department of Veterans Affairs, the United States Government and the National Institutes of Health.

The original article^1^ was updated.
