# Author Correction: Reversal of the diabetic bone signature with anabolic therapies in mice

**DOI:** 10.1038/s41413-023-00274-9

**Published:** 2023-06-13

**Authors:** Silvia Marino, Nisreen Akel, Shenyang Li, Meloney Cregor, Meghan Jones, Betiana Perez, Gaston Troncoso, Jomeeka Meeks, Scott Stewart, Amy Y. Sato, Intawat Nookaew, Teresita Bellido

**Affiliations:** 1 Department of Physiology and Cell Biology, University of Arkansas for Medical Sciences, Little Rock, AR USA; 2 Central Arkansas Veterans Healthcare System, John L. McClellan Little Rock, Little Rock, AR USA; 3 Department of Biostatistics, University of Arkansas for Medical Sciences, Little Rock, AR USA; 4 Department of Biomedical Informatics, University of Arkansas for Medical Sciences, Little Rock, AR USA; 5 Winthrop P. Rockefeller Cancer Institute, University of Arkansas for Medical Sciences, Little Rock, AR USA

**Keywords:** Bone quality and biomechanics, Pathogenesis

Correction to: *Bone Research* 10.1038/s41413-023-00261-0, published online 19 April 2023

Following publication of this article,^1^ it was reported that the name of author Scott Stewart has been mistakenly typed as Scott Stuart.

The correct name is Scott Stewart.

The original article^1^ was updated.
