# Supplementary material for: Reversal of the diabetic bone signature with anabolic therapies in mice
Source: Bone Res. 2023 Apr 19;11:19. doi: 10.1038/s41413-023-00261-0 (PMC10115794; doi:10.1038/s41413-023-00261-0)
Supplement: Supplementary file 1 — Supplemental tables [file 41413_2023_261_MOESM1_ESM.docx]

**Supplementary Tables**

**Supplementary Table S1: Femoral bone structural parameters determined by micro-CT and mechanical properties of the femoral mid-diaphysis of T2-DM mice treated with PTH or ABL.**

|  | **Control** | | **T2-DM** | | | |
| --- | --- | --- | --- | --- | --- | --- |
|  | **Veh** | | **Veh** | **PTH_100_** | **ABL_47.5_** | **ABL_95_** |
| **Distal metaphysis** |  | |  |  |  |  |
| BV/TV (%) | 5.42±1.05 | | 4.39±0.88**^** | 5.44±1.17 | 7.30±2.01***Ϯ^** | 6.21±1.66***** |
| Tb.Th (µm) | 33.66±3.67 | | 34.05±4.16 | 46.16±3.63***^** | 44.69±6.23***^** | 42.97±4.36***^** |
| Tb.N (mm^-1^) | 4.10±0.27 | | 3.84±0.31**^** | 3.48±0.27***^** | 3.53±0.20***^** | 3.42±0.32***^** |
| Tb.Sp (µm) | 242.5±16.3 | | 253.3±17.4 | 290.9±23.1***^** | 281.7±10.5***^** | 288.4±19.0***^** |
| Conn D. (mm^-3^) | 66.21±24.20 | | 55.26±17.23 | 57.21±20.77 | 103.9±28.48***Ϯ^** | 103.41±49.9***Ϯ^** |
| SMI | 2.72±0.25 | | 2.69±0.30 | 2.92±0.36 | 2.72±0.37 | 2.88±0.39 |
|  |  | |  |  |  |  |
| **Mid-diaphysis** |  | |  |  |  |  |
| Ct. Th (µm) | 189.9±9.6 | | 177.6±8.7**^** | 208.5±6.8***^** | 211.4±7.4***^** | 207.9±8.3***^** |
| BA/TA | 44.84±2.42 | | 42.77±1.16**^** | 49.16±1.22***^** | 48.96±2.05***^** | 49.74±2.32***^** |
| BA (mm^2^) | 0.97±0.06 | | 0.93±0.05 | 1.07±0.04***^** | 1.13±0.05***Ϯ^** | 1.15±0.08***Ϯ^** |
| MA (mm^2^) | 1.19±0.07 | | 1.24±0.09 | 1.11±0.07***** | 1.18±0.09 | 1.16±0.10 |
| TA (mm^2^) | 2.16±0.08 | | 2.16±0.14 | 2.18±0.10 | 2.31±0.11***Ϯ^** | 2.31±0.14***Ϯ^** |
| pMOI (mm^4^) | 0.82±0.06 | | 0.80±0.11 | 0.87±0.09 | 0.93±0.10***^** | 0.92±0.13***** |
| Porosity (%) | 3.65±0.34 | | 4.27±0.53**^** | 3.84±0.36***** | 3.81±0.34***** | 3.75±0.33***** |
|  |  | |  |  |  |  |
| **Structural properties (extrinsic)** | | |  |  |  |  |
| Ultimate force (N) | 12.6±0.81 | | 12.0±1.35 | 15.2±1.65***^** | 16.2±1.57***^** | 16.2±1.81***^** |
| Stiffness (N/mm) | 89.2±2.5 | | 74.8±9.1**^** | 91.1±16.3***** | 96.4±14.9***** | 98.5±15.1***** |
| Energy to ultimate load (mJ) | | 2.5±0.6 | 2.5±0.8 | 3.1±0.9 | 4.2±1.2***^** | 4.0±1.4***^** |
| Energy to Failure (N) | | 6.4±1.2 | 6.3±1.1 | 6.2±1.4 | 6.9±1.4 | 8.5±2.4***Ϯ^** |
|  |  | |  |  |  |  |
| **Material properties (intrinsic)** | | |  |  |  |  |
| Ultimate Stress (MPa) | 20.4±1.8 | | 19.4±2.6 | 23.2±1.5***^** | 22.7±2.2***^** | 24.0±2.2***^** |
| Young's modulus (MPa) | 1121±157 | | 1002±207 | 1097±131 | 1161±127 | 1102±229 |
| Toughness (mJ/m^3^) | 0.49±0.11 | | 0.51±0.13 | 0.57±0.18 | 0.78±0.22***Ϯ^** | 0.76±0.24***^** |

Data are presented as mean ± SD. ^p<0.05 versus control LFD mice by one way ANOVA with post hoc Dunnet’s correction for multiple comparisons; *p < 0.05 versus DM mice treated with vehicle and **Ϯ** p<0.05 versus DM mice treated with PTH by one way ANOVA with post hoc Tukey’s correction for multiple comparisons. BV/TV Bone Volume/Total Volume; Tb. Trabecular; Th. Thickness; N. Number; Sp. Separation; Conn D. Connectivity Density; SMI Structural Model Index; M.D Material Density; BA/TA cortical bone area; Ct. Cortical; B. Bone; M. Medullary; T. Tissue; pMOI Polar moment of inertia.

**Supplementary Table S2:** **Vertebral bone structural parameters determined by micro-CT and mechanical properties of the L6 vertebrae of T2-DM mice treated with PTH or ABL.**

|  | **Control** | | **T2-DM** | | | |
| --- | --- | --- | --- | --- | --- | --- |
|  | **Veh** | | **Veh** | **PTH_100_** | **ABL_47.5_** | **ABL_95_** |
| **Vertebra (L6)** |  | |  |  |  |  |
| BV/TV (%) | 19.55 ±2.45 | | 18.72±2.72 | 26.79±2.53***^** | 26.45±2.81***^** | 26.67±3.29***^** |
| Tb.Th (µm) | 41.92±2.25 | | 41.32±3.84 | 51.35±2.72***^** | 50.22±3.57***^** | 52.73±4.04***^** |
| Tb.N (mm^-1^) | 5.07±0.27 | | 5.07±0.16 | 5.41±0.25***^** | 5.31±0.25***^** | 5.36±0.3***^** |
| Tb.Sp (µm) | 191.59±11.5 | | 190.0±7.45 | 173.8±9.7***^** | 177.7±8.9***^** | 178.1±10***^** |
| Conn D. (mm^-3^) | 231.7±34.1 | | 232±15.4 | 325.7±39.2***^** | 283.4±43.8***^** | 313.58±32.1***^** |
| SMI | 0.97±0.23 | | 1.05±0.26 | 0.57±0.26***^** | 0.61±0.31***^** | 0.62±0.38***^** |
| Ct.Th (µm) | 51.2±7.6 | | 40.1±6.8^ | 43.5±5.5^ | 44.9±8.5 | 48.6±5.2* |
| Vertebral body CSA (mm^2^) | 0.43 ± 0.04 | | 0.38 ± 0.03^ | 0.42 ± 0.03***** | 0.45 ± 0.04***** | 0.47 ± 0.05***Ϯ** |
|  |  | |  |  |  |  |
| **Structural properties (extrinsic)** | | |  |  |  |  |
| Ultimate force (N) | 22.8 ± 8.1 | | 25.1 ± 13.1 | 34.8 ± 12.7**^** | 24.7 ± 10.4 | 31.6 ± 13.3 |
| Stiffness (N/mm) | 142.3 ± 44.2 | | 151.3 ± 62.9 | 169.3 ± 60.8 | 121.9 ± 49.3 | 139.8 ± 41.5 |
| Energy to ultimate load (mJ) | | 3.3 ± 1.9 | 4.1 ± 2.8 | 6.6 ± 3.0**^** | 3.8 ± 2.0 | 6.4 ± 3.7**^** |
| Energy to yield (mJ) | | 1.6 ± 1.2 | 2.3 ± 1.8 | 3.3 ± 2.1 | 1.9 ± 0.8 | 4.2 ± 3.2**^** |
|  |  | |  |  |  |  |
| **Material properties (intrinsic)** | | |  |  |  |  |
| Ultimate Stress (MPa) | 5.0 ± 1.7 | | 3.5 ± 1.9 | 3.1 ± 1.5 | 2.4 ± 1.3 | 2.6 ± 1.2 |
| Young's modulus (MPa) | 207.1 ± 72.4 | | 237.8 ± 106.9 | 262.0 ± 105.0 | 169.2 ± 58.8**Ϯ** | 190.6 ± 55.1 |
| Toughness (mJ/m^3^) | 10.8 ± 4.8 | | 9.8 ± 6.9 | 12.0 ± 5.9 | 9.4 ± 6.7 | 10.9 ± 6.4 |

Data are presented as mean ± SD. ^p<0.05 versus control LFD mice by one way ANOVA with post hoc Dunnet’s correction for multiple comparisons; *p < 0.05 versus DM mice treated with vehicle and **Ϯ** p<0.05 versus DM mice treated with PTH by one way ANOVA with post hoc Tukey’s correction for multiple comparisons. BV/TV Bone Volume/Total Volume; Tb. Trabecular; Th. Thickness; N. Number; Sp. Separation; Conn D. Connectivity Density; SMI Structural Model Index; M.D Material Density; BA/TA cortical bone area; Ct. Cortical; B. Bone; M. Medullary; T. Tissue; pMOI Polar moment of inertia.

**Supplementary Table S3:** **Femoral bone structural parameters determined by micro-CT and mechanical properties of the femoral mid-diaphysis of healthy and T2-DM mice treated with Scl-Ab.**

|  | **Control** | | | | | | **T2D** | |
| --- | --- | --- | --- | --- | --- | --- | --- | --- |
|  | **Veh** | | | **Scl-Ab** | | | **Veh** | **Scl-Ab** |
| **Distal metaphysis** |  | | |  | | |  |  |
| BV/TV (%) | 7.35±1.0 | | | 19.43±2.98***** | | | 5.68±1.19 | 17.93±3.48***** |
| Tb.Th (µm) | 40.90 ±3.34 | | | 71.42±4.06***** | | | 39.18±2.96 | 65.87±4.85***** |
| Tb.N (mm^-1^) | 4.14±0.34 | | | 4.19±0.31 | | | 3.95±0.20 | 4.36±0.22***** |
| Tb.Sp (µm) | 238.22±19.62 | | | 223.14±20.97 | | | 250.36±13.79 | 215.50±12.82***** |
| ConnD. (mm^-3^) | 57.85±12.06 | | | 88.09±14.05***** | | | 38.63±16.26**^** | 85.52±10.08***** |
| SMI | 2.78±0.20 | | | 2.01±0.30***** | | | 3.04±0.26**^** | 2.28±0.29***** |
|  |  | | |  | | |  |  |
| **Mid-diaphysis** |  | | |  | | |  |  |
| Ct. Th (µm) | 208.5±5.3 | | | 265.1±8.9***** | | | 189.8±10.6**^** | 256.8±13.0***** |
| BA/TA | 47.64±0.92 | | | 56.23±1.33***** | | | 45.31±1.03**^** | 56.47.63±1.56***** |
| BA (mm^2^) | 1.04±0.06 | | | 1.30±0.07***** | | | 0.94±0.06**^** | 1.25±0.09**^** |
| MA (mm^2^) | 1.15±0.09 | | | 1.01±0.06***** | | | 1.12±0.07 | 0.96±0.06***** |
| TA (mm^2^) | 2.19±0.15 | | | 2.31±0.12***** | | | 2.05±0.11**^** | 2.30±0.13***** |
| pMOI (mm^4^) | 0.82±0.09 | | | 0.92±0.06***** | | | 0.75±0.08 | 0.85±0.10***** |
| Porosity (%) | 4.07±0.22 | | | 3.52±0.19***** | | | 4.34±0.33**^** | 3.72±0.31***** |
|  |  | | |  | | |  |  |
| **Structural properties (extrinsic)** | | | | |  |  |  |  |
| Ultimate force (N) | 16±2.1 | | | 25.9±2.5***** | | | 15.4±2.4 | 24.2±4.9***** |
| Stiffness (N/mm) | 111±12.6 | | | 142±24.9***** | | | 90.5±15**^** | 128±28.7***** |
| Energy to ultimate load (mJ) | | 2.8±0.7 | | 5.2±0.9***** | | | 2.7±0.4 | 5.2±1.2***** |
| Energy to failure (N) | | 5.8±2.2 | | 8.9±2.7***** | | | 4.9±1.9 | 10±3.4***** |
|  | |  | |  | | |  |  |
| **Material properties (intrinsic)** | | |  |  | | |  |  |
| Ultimate Stress (MPa) | | 25.0±2.6 | | 37.6±6.5***** | | | 26.2±5.3 | 36.5±8.7***** |
| Young's modulus (MPa) | | 1433±226 | | 1576±336 | | | 1296±315 | 1531±392***** |
| Toughness (mJ/m^3^) | | 0.53±0.10 | | 0.91±0.24***** | | | 0.54±0.10 | 1.0±0.3***** |

Data are presented as mean ± SD. ^p<0.05 versus control LFD mice and *p<0.05 versus respective vehicle treated mice by two way ANOVA with post hoc Bonferroni’s correction for multiple comparisons.

**Supplementary Table S4:** **Vertebral bone structural parameters determined by micro-CT in L6 vertebrae of healthy and T2-DM mice treated with Scl-Ab.**

|  | **Control** | | **T2D** | |
| --- | --- | --- | --- | --- |
|  | **Veh** | **Scl-Ab** | **Veh** | **Scl-Ab** |
| **Vertebra (L6)** |  |  |  |  |
| BV/TV (%) | 27.14±2.45 | 47.36±3.14***** | 24.63±2.20**^** | 45.23±3.74***** |
| Tb.Th (µm) | 48.58 ±3.29 | 82.77±4.01***** | 46.94±1.46 | 78.18±6.25***** |
| Tb.N (mm^-1^) | 5.87±0.41 | 6.36±0.24 | 5.50±0.35**^** | 6.21±0.19***** |
| Tb.Sp (µm) | 163.5±14.37 | 143.8±8.47 | 173.87±12.09 | 150.31±6.98***** |
| SMI | 0.60±0.15 | -0.76±0.33***** | 0.62±0.14 | -0.67±0.37***** |
| Ct.Th (µm) | 57.9±8.6 | 86.7±10***** | 51.1±9.6 | 76.8±9.3***^** |
| Vertebral body CSA (mm^2^) | 0.36±0.03 | 0.61±0.084***** | 0.32±0.031 | 0.51±0.056***** |

Data are presented as mean ± SD. ^p<0.05 versus control LFD mice and *p<0.05 versus respective vehicle treated mice by two way ANOVA with post hoc Bonferroni’s correction for multiple comparisons.
