# Supplementary material for: Reversal of the diabetic bone signature with anabolic therapies in mice
Source: Bone Res. 2023 Apr 19;11:19. doi: 10.1038/s41413-023-00261-0 (PMC10115794; doi:10.1038/s41413-023-00261-0)
Supplement: Supplementary file 2 — Supplemental Figures [file 41413_2023_261_MOESM2_ESM.pdf]

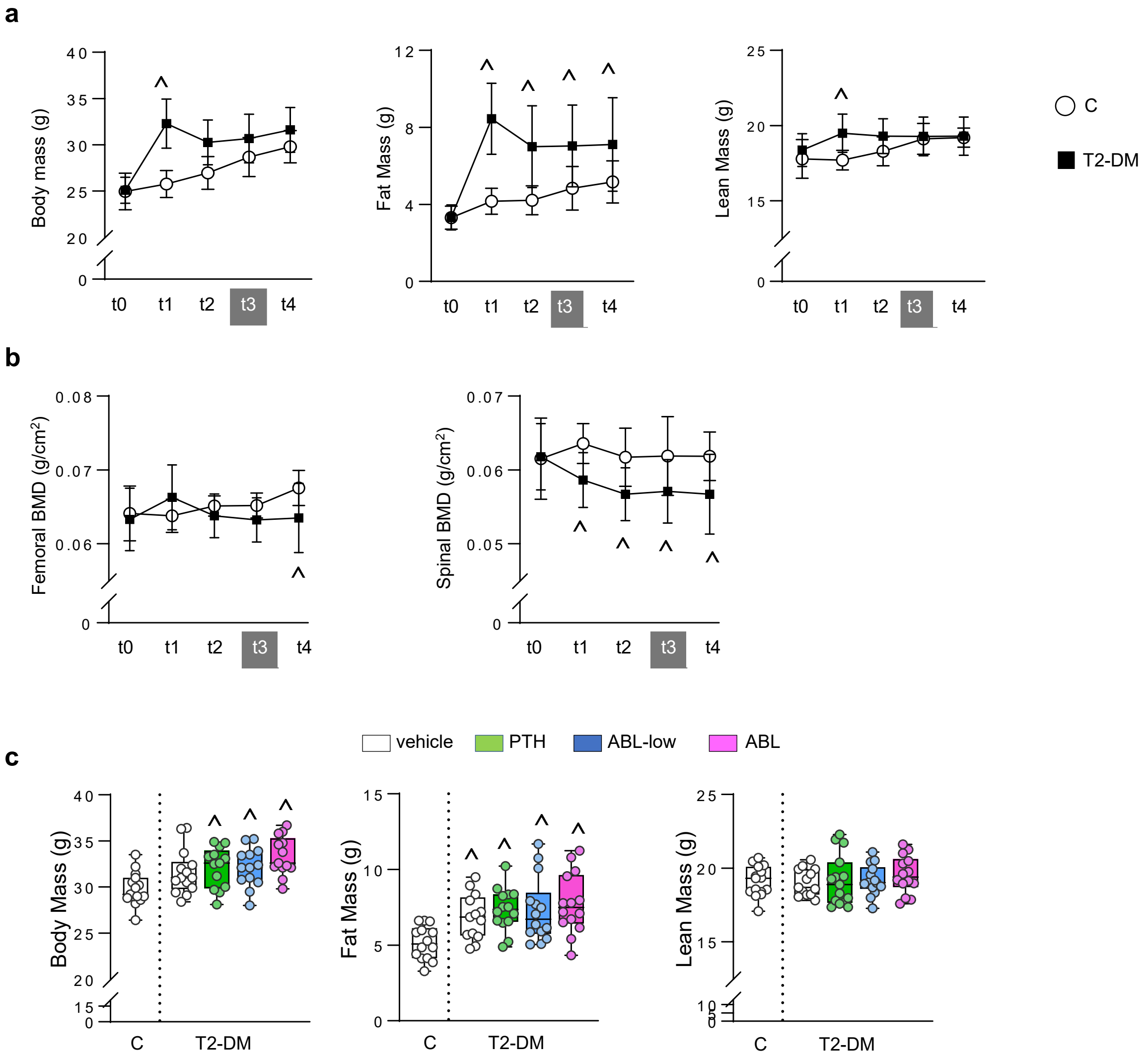

Supplementary Figure 1

Longitudinal analysis of **a** body mass, fat mass and lean mass and **b** femoral and spinal bone mineral density (BMD). **c** Quantification of body, fat and lean mass after (t4) treatment with PTH or ABL. n=12-15 mice/group. Data are presented as box & whiskers plots where each dot represents a mouse. ^p<0.05 versus control LFD mice by one way ANOVA with post hoc Dunnet's correction for multiple comparisons; *p<0.05 versus DM mice treated with vehicle by one way ANOVA with post hoc Tukey's correction.

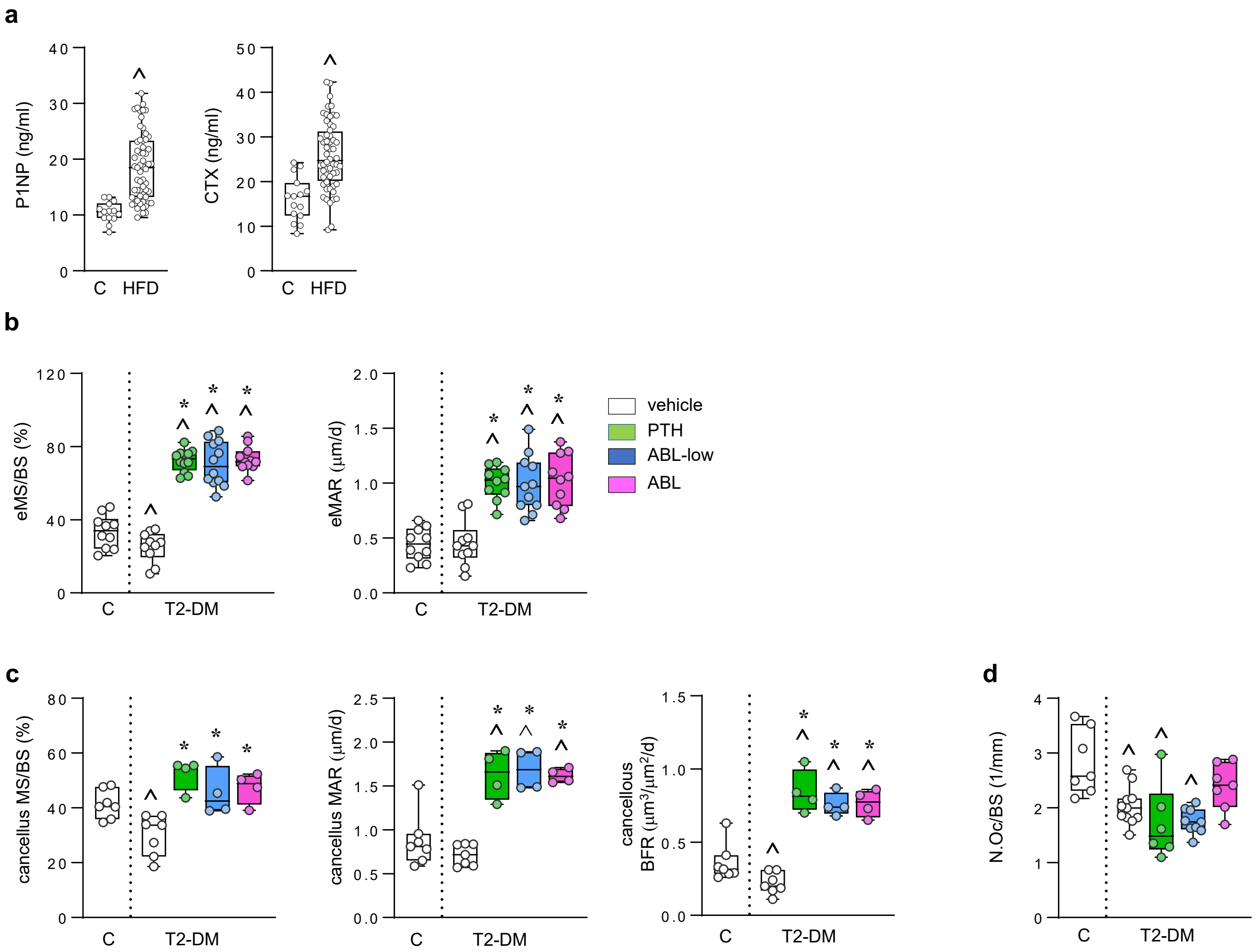

Supplementary Figure 2

a Serum P1NP and CTX measure after 4 weeks of HFD. Dynamic bone histomorphometric analysis of mineralized surface/bone surface (MS/BS) and mineral apposition rate (MAR) at **b** the endosteal surface and **c** cancellous surface measured after (t4) treatment with PTH or ABL. **d** Osteoclast number per bone surface (NOc/BS) measured after (t4) treatment with PTH or ABL. n=12-15 mice/group. Data are presented as box & whiskers plot where each dot represents a mouse. ^p<0.05 versus C mice by by unpaired and two-tailed Student's t-test (**a**) or one-way ANOVA with post hoc Dunnet's correction (**b**); *p<0.05 versus T2-DM mice treated with vehicle and ↑p<0.05 versus T2-DM mice treated with PTH by one way ANOVA with post hoc Tukey's correction.

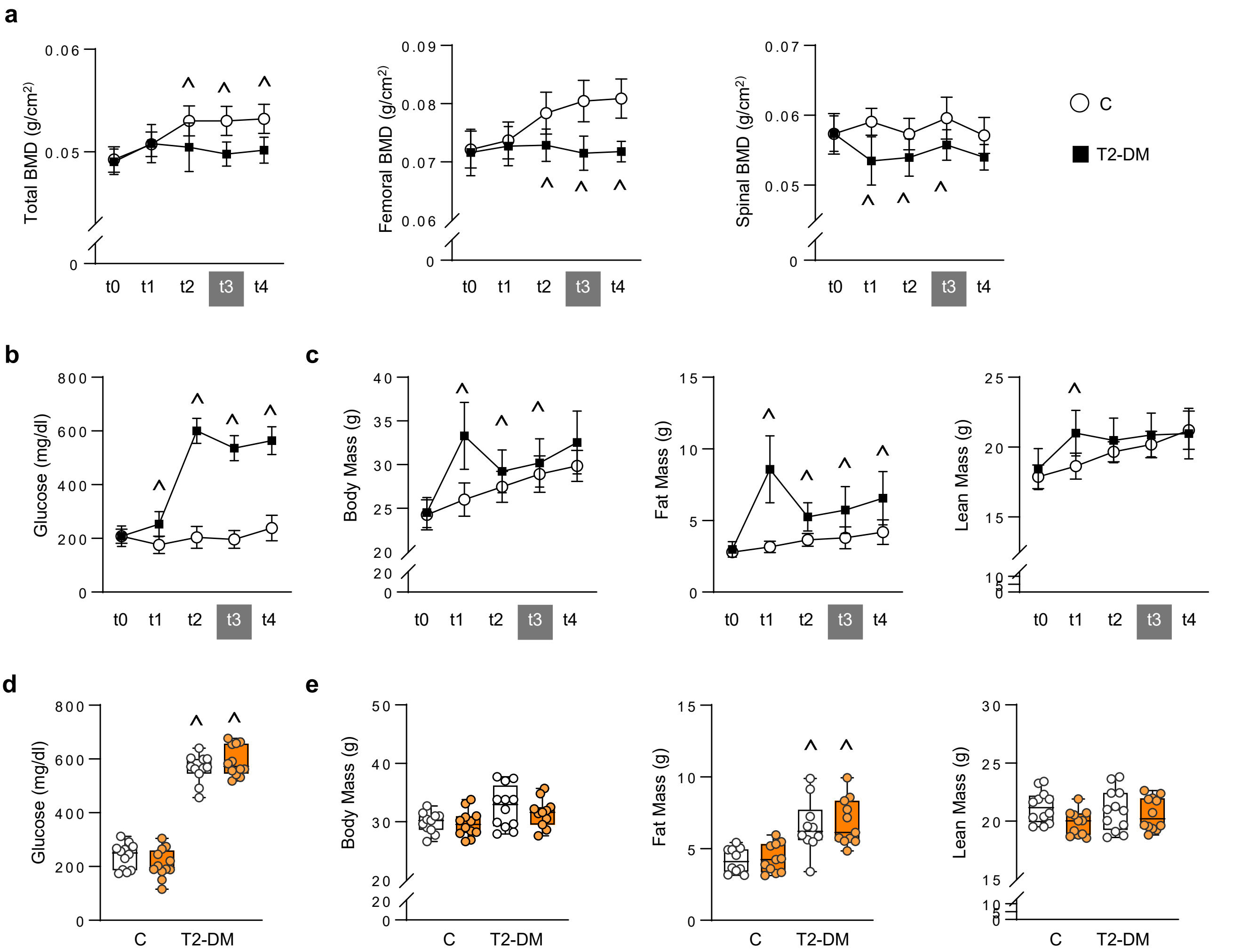

Supplementary Figure 3

Longitudinal analysis of **a** total, femoral and spinal bone mineral density (BMD) **b** blood glucose and **c** body weight, fat mass and lean mass from the Scl-Ab experiment. Quantification of **d** glucose levels and **e** body, fat and lean mass after (t4) treatment with Scl-Ab. For all analyses, n=10-12 mice/group. Data are presented as box & whiskers plot where each dot represents a mouse. ^p<0.05 versus control LFD mice and *p<0.05 versus respective vehicle treated mice by two-way ANOVA with post hoc Bonferroni's correction for multiple comparisons

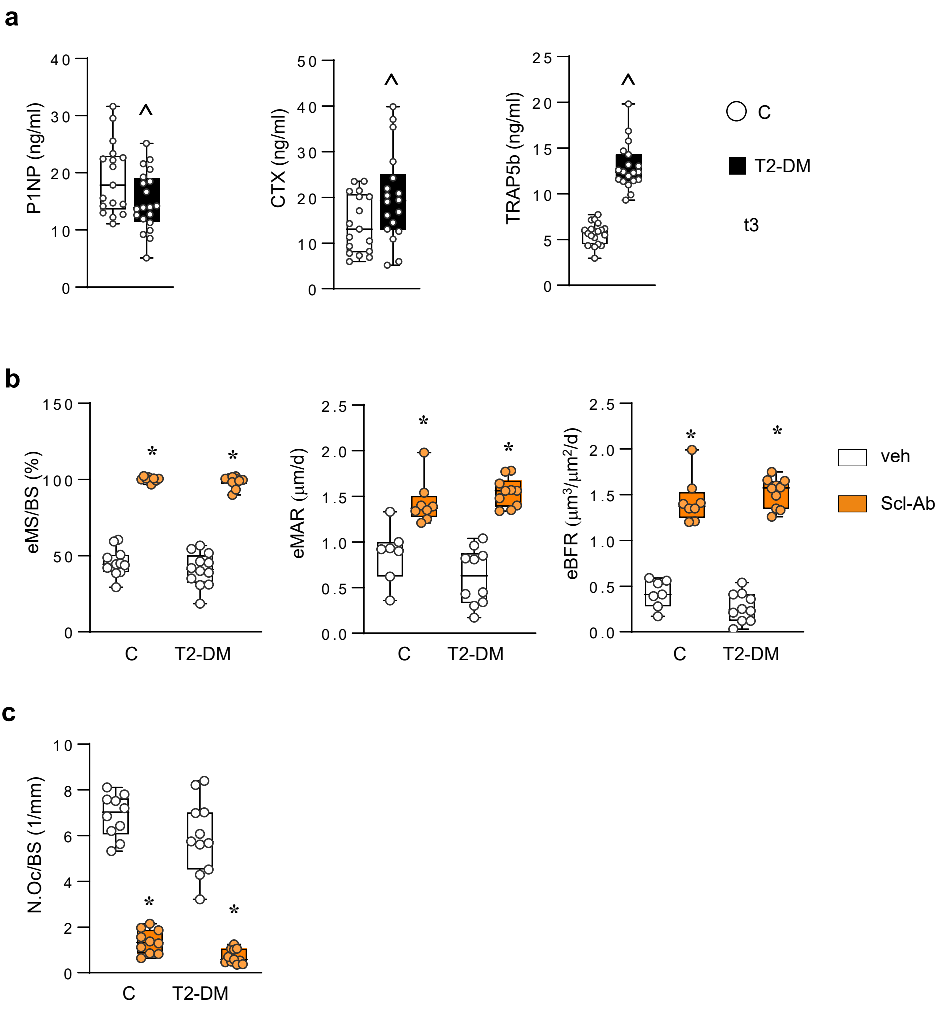

Supplementary Figure 4

a Serum P1NP, CTX and TRAP5b measure before (t3) treatment with Scl-Ab. **b** Dynamic bone histomorphometric analysis of mineralized surface/bone surface (MS/BS), mineral apposition rate (MAR) bone formation rate (BFR) at the endosteal surface measured after (t4) treatment with vehicle or Scl-Ab. **c** Osteoclast number per bone surface (N.Oc/BS) measure after (t4) treatment with vehicle or Scl-Ab. n=10-12 mice/group. Data are presented as box & whiskers plot where each dot represents a mouse. ^p<0.05 versus control LFD mice by unpaired and two-tailed Student's t-test (**a**) or by two-way ANOVA with post hoc Bonferroni's correction for multiple comparisons (**b**); *p<0.05 versus respective vehicle-treated mice by two-way ANOVA with post hoc Bonferroni's correction for multiple comparisons.
